# Impact of self-reported race on Villalta Scale postthrombotic syndrome scores and correlation with venous disease-specific quality of life: an exploratory analysis of the Acute Venous Thrombosis: Thrombus Removal with Adjunctive Catheter-Directed Thrombolysis Trial

**DOI:** 10.1016/j.rpth.2024.102609

**Published:** 2024-10-29

**Authors:** James Shih, Chu-Shu Gu, Suresh Vedantham, John Kaufman, Susan R. Kahn

**Affiliations:** 1 Department of Medicine, McGill University, Montreal, Quebec, Canada; 2 Centre for Regulatory Excellence, Statistics and Trial, Ottawa, Ontario, Canada; 3 Washington University School of Medicine, St Louis, Missouri, USA; 4 Oregon Health and Science University, Portland, Oregon, USA; 5 Centre of Excellence in Thrombosis and Anticoagulation Care, Jewish General Hospital, Montreal, Quebec, Canada

**Keywords:** ethnicity, postthrombotic syndrome, racial groups, venous thrombosis

## Abstract

**Background:**

The Villalta Scale (VS) to diagnose postthrombotic syndrome (PTS) consists of 5 patient-reported leg symptoms and 6 clinician-rated leg signs. It is unknown how the scale performs across racial groups.

**Objectives:**

Our study explored if there were differences in VS scores, particularly clinician-rated signs components, according to self-reported race.

**Methods:**

Exploratory analysis of the ATTRACT trial, a randomized controlled trial conducted at 56 US sites that investigated pharmacomechanical catheter-directed thrombolysis to prevent PTS after proximal deep vein thrombosis (DVT). At the 6-month visit after randomization, we compared self-reported Black (n = 123) and White (n = 541) participants for mean total VS score, VS symptoms score, VS signs score, individual signs scores, and correlation coefficients between VS signs and VS symptoms scores and between VS signs and Venous Insufficiency Epidemiological and Economic Study Quality of Life (VEINES-QOL) scores (a self-reported venous disease-specific quality of life measure).

**Results:**

Mean total VS score (4.67 vs. 4.12, *P* = .54),VS signs score (1.66 vs. 2.00, *P* = .07), and VS symptoms score (2.83 vs. 2.04, *P* = .10) were similar between Black and White participants. The mean score for one individual VS sign, venous ectasia, was lower in Black vs. White participants (0.24 vs. 0.63, *P*< .01). There was similar, modest correlation in Black and White participants between VS signs and VS symptoms scores (*r*_black_ = 0.19; *r*_white_ = 0.23) and between VS signs and VEINES-QOL scores (*r*_black_ = −0.32; *r*_white_ = −0.30). Results were adjusted for ATTRACT trial treatment group, age, sex, body mass index, DVT extent, hypertension, diabetes, dyslipidemia, and congestive heart failure.

**Conclusion:**

The findings suggest that some differences in VS scores exist according to self-reported race. It is unclear whether these reflect clinicians’ underrating of some VS signs and/or differences in PTS severity. Further work is needed to understand how the VS performs across racial groups.

## Essentials

• Postthrombotic syndrome (PTS) is a common complication following deep vein thrombosis.
• In the ATTRACT trial population, we compared Villalta Scale (VS) by self-reported race.
• VS were similar, but venous ectasia scores were lower in Black vs. White participants.
• VS scores may differ according to self-reported race, which merits further study.

## 1 Introduction

Postthrombotic syndrome (PTS) describes the constellation of symptoms and signs that develop in 20% to 50% of patients after a deep vein thrombosis (DVT) [1]. PTS is associated with significant functional burden and reduced quality of life [2].

The diagnosis of PTS is above all clinical, relying on the presence of characteristic symptoms and signs. These features have been incorporated into various scoring systems in an effort to standardize the definition of PTS [3]. One such scoring system is the Villalta Scale (VS), which consists of 5 patient-reported symptoms (pain, cramps, paresthesia, pruritus, and heaviness) and 6 clinician-rated signs (edema, skin induration, hyperpigmentation, pain with calf compression, venous ectasia, and redness) [4]. The International Society on Thrombosis and Haemostasis recommends use of the VS as a standard to diagnose and rate the severity of PTS given its well-established interrater reliability, practicality, and external validity [5,6].

Although there is evidence to support the general reliability of the VS, it is unclear whether it adequately describes PTS in patients of different self-reported race. Previous clinical trials that have used the VS to diagnose PTS have had minimal inclusion or reporting of individuals of differing racial or ethnic backgrounds. For example, in the Ultrasound-Accelerated Catheter-Directed Thrombolysis on Preventing Post-Thrombotic Syndrome (CAVA) trial, the race of the participants was not reported [7]. The Compression Stockings to Prevent the Post-Thrombotic Syndrome (SOX) trial did include self-reported race as a demographic variable; however, the trial participants were predominantly White (>90%) [8].

In the Acute Venous Thrombosis: Thrombus Removal with Adjunctive Catheter-Directed Thrombolysis (ATTRACT) trial, there was detailed information about the self-reported race of participants; participants self-identified as a variety of races, including Black/African American, Asian, American Indian/Alaskan Native, or Native Hawaiian/Other Pacific Islander [9]. Thus, the purpose of this study was to perform a post hoc exploratory analysis of the ATTRACT trial to explore whether differences existed in PTS scores according to self-reported race.

## 2 Methods

### 2.1 ATTRACT trial methods

The ATTRACT trial was a phase 3, open-label randomized controlled trial conducted in 56 centers in the United States from 2009-2014. Patients with an acute symptomatic DVT in the femoral, common femoral, or iliac vein were assigned to receive pharmacomechanical catheter-directed thrombolytic therapy (PCDT) plus standard care or standard care alone (no PCDT). All patients received anticoagulation as well as elastic compression stockings. Patients in the PCDT arm received infusions of recombinant tissue plasminogen activator directly into the thrombus-containing vessel, with adjunctive procedures such as balloon maceration, thrombectomy, angioplasty, and stent placement performed at the discretion of the treating physician.

Trial participants were assessed at follow-up study visits at 10 days and 1, 6, 12, 18, and 24 months post randomization. The primary outcome was development of PTS in the 6 to 24 month follow-up period post randomization. The VS in the DVT-affected leg was used to identify the incidence and characterize the severity of PTS. Each component of the VS was rated on a scale of 0 (absent) to 3 (severe). A total score of ≥5 denoted PTS; scores of 5 to 9, 10 to 14, and ≥15 (or presence of a venous ulcer) denoted mild, moderate, and severe PTS, respectively.

The Venous Insufficiency Epidemiological and Economic Study Quality of Life (VEINES-QOL) measure, a venous disease-specific instrument that has been previously validated for describing quality of life outcomes in patients with DVT and PTS [10], was administered at baseline and at all follow-up visits. Lower scores indicate worse quality of life.

As part of the baseline demographic information collected in the ATTRACT trial, race as self-reported by trial participants was documented as White, Black/African American, Asian, American Indian/Alaskan Native, Native Hawaiian/Other Pacific Islander, or “Not reported or refused.” The trial protocol was approved by the ethics committee of each participating center, and informed consent was obtained from all participants. Further description of the ATTRACT trial design and protocol can be found in the primary publication.

### 2.2 Exploratory analysis methods

In this study, 2 analysis groups were considered: Black or White participants. As the number of participants who self-identified as the other races was too small for meaningful statistical analyses, these were not included in this exploratory analysis.

We compared demographic and other baseline variables in Black vs. White participants. Analyses of VS and VEINES-QOL scores were conducted using data from the 6-month timepoint after randomization, as based on the ATTRACT trial data; this was the timepoint at which most incident cases of PTS had occurred. If VS or VEINES-QOL scores were not available at the 6-month timepoint, corresponding data from the 12-month timepoint were imputed instead. Missing data are detailed in Supplementary Table 1.

Mean scores for each of the 5 patient-reported and 6 clinician-rated components of the VS were compared in self-identified Black and White participants. Similar comparisons were made for mean total VS score, mean total patient-reported symptoms scores, and mean total clinician-rated signs scores. Given the underlying distribution of the VS scores were nonnormal and asymmetrical, nonparametric statistical tests were used. Using Wilcoxon tests stratified by the treatment group, corresponding *P* values were calculated for each comparison, with statistical significance set at *P* < .05. The statistical significances of the distributions of score severity for individual VS signs and symptoms were calculated using generalized Cochran-Mantel-Haenszel tests, controlling for the treatment group, with statistical significance set at *P* < .05.

As in the original ATTRACT trial, the mean VS score from 6 to 24 months was lower in the treatment (ie, PCDT) group, we adjusted for treatment group (ie, PCDT or no PCDT) in our analyses.

To assess the relationship between patient-reported symptoms scores and clinician-rated signs scores, partial Spearman’s rank correlation testing was performed to generate correlation coefficients, denoted *r* in the analyses. These coefficients range from −1 to 1, with 1 being a perfect positive correlation, −1 a perfect negative correlation, and 0 denoting no correlation. Correlation coefficients were calculated between each of the 5 patient-reported symptoms scores of the VS and each of the 6 clinician-rated signs scores, between each of the 5 patient-reported VS symptoms scores and the total clinician-rated VS signs score, and between each of the 6 individual clinician-rated VS signs scores and the total patient-reported VS symptoms scores. Analyses were adjusted for ATTRACT treatment groups (PCDT or no PCDT), baseline demographic variables (age, sex, and body mass index), extent of DVT, and history of hypertension, diabetes, high cholesterol, and heart failure. Correlation coefficients were deemed to be strong if their absolute value was >0.5, moderate if between 0.3 and 0.5, and weak if between 0 and 0.3. Statistical significance was determined if the coefficients were significantly different from zero (*P* < .05). Analyses were conducted separately for Black and White participants.

As an alternate indicator of PTS severity as reflected by venous disease-specific quality of life, adjusted correlation coefficients were also calculated between the 6 VS signs scores and VEINES-QOL scores in Black and White participants.

Statistical analyses were performed using SAS software version 9.4.

## 3 Results and Discussion

Six hundred ninety-one participants were randomized in the ATTRACT trial. Of these, 541 self-identified their race as White (78%), 123 participants self-identified as Black (18%), and 27 participants self-identified as Other (4%). The distribution of White and Black participants in the PCDT and No PCDT intervention arms was similar (PCDT: 265 White participants, 61 Black participants; No PCDT: 276 White participants, 62 Black participants). Baseline demographic and other characteristics were similar in Black and White participants (Table 1).

**Table 1:** Baseline characteristics by self-reported race.

Characteristic No. of subjects	Black participants	White participants
	n = 123	n = 541
**Age, y**		
n, mean (SD)	123, 50 (14)	540, 51 (13)
min, Q1, median, Q3, max	16, 41, 52, 61, 75	16, 42, 53, 62, 75
**Age group,** n (%)		
<40	30 (24)	115 (21)
40–64	71 (58)	330 (61)
65–75	22 (18)	95 (18)
Unknown	0	1 (<1)
**Sex,** n (%)		
Female	44 (36)	211 (39)
Male	79 (64)	330 (61)
**BMI, kg/m**^**2**^		
n, mean (SD)	121, 32.2 (8.2)	539, 31.7 (7.4)
min, Q1, median, Q3, max	14.8, 26.4, 30.3, 37.5, 55.2	18.4, 26.9, 30.6, 35.1, 68.0
**BMI class:** n (%)		
<25 kg/m^2^	20 (16)	91 (17)
25 to <30 kg/m^2^	39 (32)	161 (30)
≥30 kg/m^2^	62 (50)	287 (53)
Unknown	2 (2)	2 (<1)
**Extent of DVT,** n (%)		
Iliofemoral DVT	68 (55)	306 (57)
Femoropopliteal DVT	55 (45)	235 (43)
**Comorbid conditions**^a^**,** n (%)		
Hypertension	57 (46)	215 (40)
Diabetes	29 (24)	78 (14)
High cholesterol	38 (31)	154 (28)
Congestive heart failure	7 (6)	21 (4)

BMI, body mass index; DVT, deep vein thrombosis.

a Subject may fit into more than one category.

The mean (SD) total VS score was similar in Black and White participants (4.67 [4.88] vs. 4.12 [4.41], *P* =.54). Similarly, the mean (SD) total clinician-rated VS sign score was similar in Black and White participants (1.66 [2.44] vs. 2.00 [2.52], *P* = .07), as was the mean (SD) total patient-reported VS symptoms score (2.83 [3.61] vs. 2.04 [2.72], *P* = .10). Two of the 5 patient-reported symptoms demonstrated a higher mean (SD) score in Black participants (pain 0.79 [0.97] vs. 0.51 [0.80], *P* = .01; pins and needles 0.54 [0.91] vs. 0.34 [0.67], *P* = .05). The mean scores of the 3 other patient-reported symptoms were not significantly different between Black and White participants (Table 2). For the 6 clinician-rated VS signs, the mean (SD) venous ectasia score was lower in Black participants (0.24 [0.61] vs. 0.63 [0.83], *P* < .01). There were no significant differences in the mean scores of the remaining 5 clinician-rated VS signs between Black and White participants (Table 2).

**Table 2:** Villalta Scale total scores, total patient-reported symptoms scores, and total clinician-rated signs scores at 6 months by self-reported race.

Characteristic No. of subjects	Black participants	White participants	*P*
	n = 123	n = 541	
**Villalta Scale total score**	100, 4.67 (4.88)	473, 4.12 (4.41)	.54^a^
n, mean (SD)			
**Villalta Scale score, symptoms**	101, 2.83 (3.61)	475, 2.04 (2.72)	.10^a^
n, mean (SD)			
**Cramps,** n (%)			.07^b^
No/minimal	71 (70)	338 (71)	
Mild	19 (19)	89 (19)	
Moderate	4 (4)	36 (8)	
Severe	7 (7)	11 (2)	
*n,* mean (SD)	101, 0.48 (0.87)	474, 0.41 (0.73)	.72^a^
**Itching,** n (%)			.77^b^
No/minimal	74 (73)	368 (77)	
Mild	18 (18)	76 (16)	
Moderate	6 (6)	22 (5)	
Severe	3 (3)	9 (2)	
n, mean (SD)	101, 0.39 (0.73)	475, 0.31 (0.65)	.33^a^
**Pins and needles,** n (%)			.02^b^^,^^c^
No/minimal	68 (67)	360 (76)	
Mild	18 (18)	77 (16)	
Moderate	8 (8)	30 (6)	
Severe	7 (7)	8 (2)	
*n,* mean (SD)	101, 0.54 (0.91)	475, 0.34 (0.67)	.05^a^^,^^c^
**Leg heaviness,** n (%)			.15^b^
No/minimal	63 (62)	322 (68)	
Mild	18 (18)	93 (20)	
Moderate	14 (14)	49 (10)	
Severe	6 (6)	11 (2)	
*n,* mean (SD)	101, 0.63 (0.94)	475, 0.47 (0.77)	.16^a^
**Pain,** n (%)			.02^b^^,^^c^
No/ninimal	53 (52)	309 (65)	
Mild	23 (23)	104 (22)	
Moderate	18 (18)	46 (10)	
Severe	7 (7)	16 (3)	
n, mean (SD)	101, 0.79 (0.97)	475, 0.51 (0.80)	.01^a^^,^^c^
**Villalta Scale score, signs**	100, 1.66 (2.44)	473, 2.00 (2.52)	0.07^a^
n, mean (SD)			
**Pretibial edema,** n (%)			.63^b^
No/minimal	69 (69)	310 (66)	
Mild	16 (16)	102 (22)	
Moderate	11 (11)	47 (10)	
Severe	4 (4)	14 (3)	
n, mean (SD)	100, 0.50 (0.85)	473, 0.50 (0.79)	.67^a^
**Skin induration,** n (%)			.46^b^
No/minimal	85 (85)	416 (88)	
Mild	9 (9)	37 (8)	
Moderate	6 (6)	16 (3)	
Severe	0	4 (1)	
n, mean (SD)	100, 0.21 (0.54)	473, 0.17 (0.51)	.40^a^
**Hyperpigmentation,** n (%)			.22^b^
No/minimal	65 (66)	356 (75)	
Mild	26 (26)	83 (18)	
Moderate	7 (7)	29 (6)	
Severe	1 (1)	5 (1)	
n, mean (SD)	99, 0.43 (0.67)	473, 0.33 (0.64)	.06^a^
**Venous ectasia,** n (%)			<.01^b^^,^^c^
No/minimal	83 (83)	265 (56)	
Mild	12 (12)	140 (30)	
Moderate	3 (3)	48 (10)	
Severe	2 (2)	20 (4)	
n, mean (SD)	100, 0.24 (0.61)	473, 0.63 (0.83)	<.01^a^^,^^c^
**Redness,** n (%)			.34^b^
No/minimal	89 (89)	385 (81)	
Mild	9 (9)	71 (15)	
Moderate	2 (2)	16 (3)	
Severe	0	1 (<1)	
n, mean (SD)	100, 0.13 (0.39)	473, 0.22 (0.50)	.07^a^
**Pain during calf compression,** n (%)			.51^b^
No/minimal	90 (90)	423 (89)	
Mild	6 (6)	36 (8)	
Moderate	4 (4)	10 (2)	
Severe	0	4 (1)	
n, mean (SD)	100, 0.14 (0.45)	473, 0.14 (0.47)	.90^a^

Villalta Scale total scores have a range of 0–33. Villalta Scale symptoms scores have a range of 0–15. Villalta Scale signs scores have a range of 0–18. Individual symptoms and signs scores have ranges of 0–3.

a *P* values for mean scores calculated from stratified Wilcoxon (Van Elteren) test, stratified by treatment group.

b *P* values for the distribution of score severities calculated from generalized Cochran-Mantel-Haenszel test controlling for treatment group.

c Statistically significant for *P* ≤ .05.

**Figure 1:**
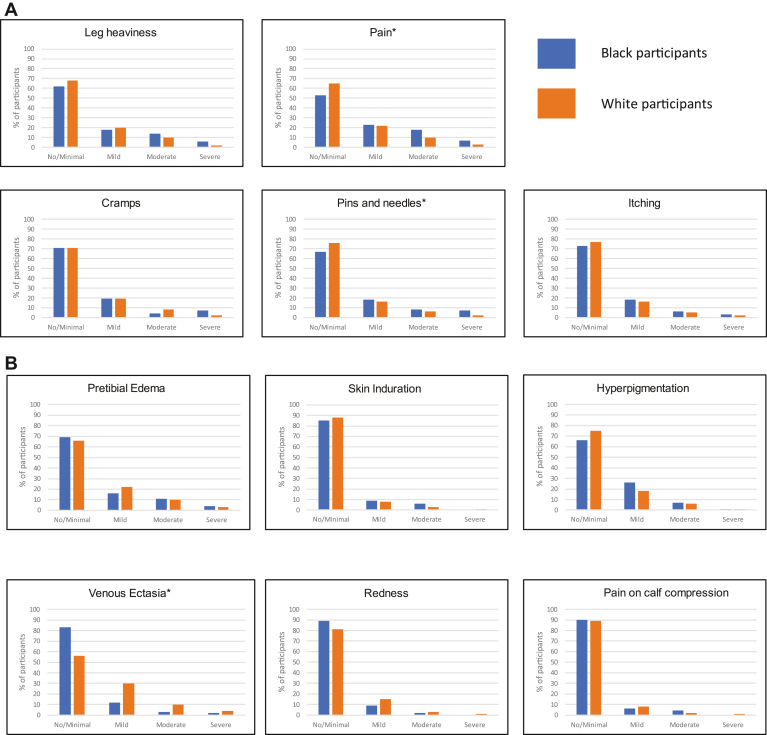
Distribution of severity of (A) patient-reported and (B) clinician-rated components of Villalta Scale scores by self-reported race. Distribution based on percentages. Asterisks indicate statistically significant differences calculated using generalized Cochran-Mantel-Haenszel tests in distribution of scores between Black (blue bars) and White (orange bars) participants.

The distribution of the severity of the patient-reported VS symptoms showed differences in Black and White participants, with higher distributions of moderate and severe categories for the symptoms pain and pins and needles in Black participants (Figure 1A). Meanwhile, the distribution of clinical signs severity was similar in Black and White participants (Figure 1B), except for venous ectasia, where a larger proportion of White participants had greater severity categories (*P* < .01).

The adjusted partial Spearman correlation coefficient between patient-reported and clinician-rated VS scores were of similar magnitude in Black and White participants; however, the correlation in Black participants was not statistically significant (*r*_black_ = 0.19; 95% CI: −0.03 to 0.40; *r*_white_ = 0.23; 95% CI: 0.14- 0.32). For both participant groups, the strengths of the correlations were modest, with *r* values <0.5 (Table 3). The correlation coefficients between scores for each individual patient-reported symptom and clinician-rated sign were similarly modest in both Black and White participants, ranging from −0.13 to 0.39 in Black and 0.02 to 0.35 in White participants. However, Black participants had fewer statistically significant correlation coefficients between VS symptoms and signs compared with White participants, in whom the majority of correlation coefficients were statistically significant (Table 3).

**Table 3:** Partial Spearman’s rank correlation coefficients^a^ (95% CI) between patient-reported and clinician-rated Villalta Scale by self-reported race.

Clinician-rated signs component score	Patient-reported symptoms component score					
	Cramps	Itchiness	Pins/needles	Leg heaviness	Pain	Total symptoms score
White participants						
Redness	0 (−0.23 to 0.24)	0.03 (−0.19 to 0.26)	0.05 (−0.16 to 0.25)	0.13 (−0.11 to 0.35)	0.13 (−0.09 to 0.35)	0.08 (−0.16 to 0.3)
Hyperpigmentation	−0.09 (−0.28 to 0.11)	0.01 (−0.18 to 0.2)	0.13 (−0.06 to 0.32)	0.07 (−0.12 to 0.26)	0.05 (−0.13 to 0.22)	0.02 (−0.16 to 0.21)
Venous ectasia	0 (−0.15 to 0.15)	−0.01 (−0.17 to 0.15)	0 (−0.15 to 0.16)	0.08 (−0.08 to 0.24)	−0.13 (−0.28 to 0.02)	−0.03 (−0.21 to 0.15)
Edema	0.11 (−0.13 to 0.33)	0.07 (−0.18 to 0.32)	0.15 (−0.08 to 0.36)	0.27 (0.01-0.49)^b^	0.34 (0.11-0.53)^b^	0.21 (−0.03 to 0.42)
Induration	−0.03 (−0.24 to 0.19)	−0.01 (−0.25 to 0.23)	−0.03 (−0.25 to 0.18)	0.14 (−0.09 to 0.36)	0.17 (−0.07 to 0.38)	0.07 (−0.17 to 0.3)
Pain on compression	0.19 (−0.06 to 0.42)	0.21 (−0.08 to 0.46)	0.14 (−0.11 to 0.38)	0.37 (0.13-0.57)^b^	0.39 (0.19-0.56)^b^	0.32 (0.11-0.50)^b^
Total Signs Score	0.02 (−0.19 to 0.24)	0.11 (−0.13 to 0.33)	0.14 (−0.07 to 0.34)	0.29 (0.07-0.48)^b^	0.25 (0.03-0.45)^b^	0.19 (−0.03 to 0.40)
Black participants						
Redness	0.13 (0.03-0.22)^b^	0.04 (−0.06 to 0.14)	0.06 (−0.04 to 0.16)	0.09 (−0.02 to 0.19)	0.10 (0-0.2)	0.10 (0-0.2)
Hyperpigmentation	0.08 (−0.02 to 0.18)	0.1 (0-0.2)	0.06 (−0.04 to 0.15)	0.12 (0.02-0.22)^b^	0.07 (−0.03 to 0.16)	0.10 (0.01-0.2)^b^
Venous ectasia	0.1 (0.01-0.19)^b^	0.14 (0.04-0.23)^b^	0.06 (−0.03 to 0.15)	0.08 (−0.01 to 0.17)	0.07 (−0.02 to 0.16)	0.1 (0-0.19)^b^
Edema	0.16 (0.06-0.26)^b^	0.15 (0.05-0.25)^b^	0.08 (−0.03 to 0.18)	0.19 (0.09-0.28)^b^	0.17 (0.07 to 0.27)^b^	0.19 (0.09-0.28)^b^
Induration	0.11 (0-0.22)^b^	0.02 (−0.08 to 0.13)	0.04 (−0.06 to 0.15)	0.11 (0.01-0.22)^b^	0.09 (−0.02 to 0.2)	0.13 (0.03-0.22)^b^
Pain on compression	0.26 (0.15-0.37)^b^	0.17 (0.05-0.28)^b^	0.24 (0.12-0.35)^b^	0.28 (0.18-0.38)^b^	0.35 (0.26-0.45)^b^	0.32 (0.23-0.40)^b^
Total Signs Score	0.2 (0.1-0.29)^b^	0.18 (0.08-0.27)^b^	0.14 (0.04-0.24)^b^	0.21 (0.12-0.3)^b^	0.2 (0.1-0.29)^b^	0.23 (0.14-0.32)^b^

VEINES-QOL, Venous Insufficiency Epidemiological and Economic Study Quality of Life.

a Adjusted for treatment group, age, sex, body mass index, extent of index deep vein thrombosis, hypertension, diabetes, high cholesterol, and congestive heart failure.

b Indicates a statistically significant *r* value, defined as being statistically different from zero (*P* < .05).

The correlation coefficients between the clinician-rated VS scores and the patient-reported VEINES-QOL scores were statistically significant in both Black and White participants, even after adjusting for treatment group, age, sex, body mass index, index of DVT, hypertension, diabetes, high cholesterol, and congestive heart failure (*r*_black_ = −0.32, 95% CI: −0.54 to −0.07; *r*_white_ = −0.24, 95% CI: −0.33 to −0.14). There was an inverse correlation between VS scores and VEINES-QOL scores, which is expected and has been previously demonstrated [10]. Accordingly, the majority of individual clinician-rated signs demonstrated weak-to-moderate correlations with VEINES-QOL scores in both Black and White participants (Table 4).

**Table 4:** Partial Spearman’s rank correlation coefficients^a^ (95% CI) between Villalta Scale clinician-rated signs scores and patient-reported VEINES-QOL scores.

Villalta Scale clinician-rated signs scores	Correlation coefficient^a^ with patient-reported VEINES-QOL scores	
	Black participants	White participants
	Estimate (95% CI)	
Total signs score	−0.32 (−0.54 to −0.07)^b^	−0.24 (−0.33 to −0.14)^b^
Redness	−0.30 (−0.49 to −0.08)^b^	−0.10 (−0.19 to 0)
Hyperpigmentation	−0.19 (−0.40 to 0.04)	−0.13 (−0.23 to −0.04)^b^
Venous ectasia	−0.04 (−0.26 to 0.18)	−0.11 (−0.21 to −0.02)^b^
Edema	−0.40 (−0.59 to −0.16)^b^	−0.20 (−0.28 to −0.10)^b^
Induration	−0.18 (−0.43 to 0.09)	−0.12 (−0.21 to −0.03)^b^
Pain on compression	−0.32 (−0.50 to −0.10)^b^	−0.30 (−0.38 to −0.20)^b^

VEINES-QOL, Venous Insufficiency Epidemiological and Economic Study Quality of Life.

a Adjusted for treatment group, age, sex, body mass index, extent of index deep vein thrombosis, hypertension, diabetes, high cholesterol, and congestive heart failure.

b Indicates a statistically significant *r* value, defined as being statistically different from zero.

Our results indicate that, in general, VS scores were similar in Black and White participants. There was no significant difference in mean total VS scores between the 2 groups. These findings suggest that the utility of the VS to assess and characterize the severity of PTS may extend to White and Black patients.

However, we identified some differences in the scores of components of the VS in Black and White participants. Black participants had lower mean scores for the clinician-rated sign venous ectasia compared with White participants while having higher mean patient-reported symptom scores for pain and pins and needles.

These differences in VS scoring in self-reported Black participants could be due to differences in the disease process itself in this population. It is possible that PTS in Black patients may manifest more severely as symptoms rather than as outward clinical signs. This is difficult to corroborate in the literature because studies on PTS in patients of differing racial or ethnic backgrounds are lacking. One subanalysis of the ATTRACT trial, which investigated risk factors for PTS occurrence, did not identify race as a risk factor [11]. However, several studies have demonstrated that the incidence and severity of DVT as well as of venous thromboembolic events in general are higher in Black patients [12,13]. Furthermore, comorbidities such as obesity and chronic inflammatory diseases are known to be predisposing risk factors for PTS [14,15]. The prevalence of obesity is higher in Black patients, as are rheumatologic conditions, eg, gout and systemic lupus erythematosus [[16], [17], [18]]. The presence of these comorbidities may have an additive effect on the pain and inflammation that Black patients may experience with PTS. Further research is needed to delineate the interplay between these conditions and the development of PTS in different racial groups.

Alternatively, the differences in VS scores we observed could suggest a possible phenomenon of underrating of clinical signs in Black patients. A large body of research has demonstrated that many classical dermatologic signs present differently in patients with skin of color (SOC). In the field of dermatology, SOC refers to individuals of racial or ethnic groups with shared dermatologic characteristics, which include increased constitutive pigmentation, propensity toward reactive pigment alteration, and high skin phototype [19]. Many of the clinician-rated features of the VS include dermatologic signs that are known to present differently in patients with SOC. For example, hyperpigmentation is often masked by background pigmentation in patients with SOC, and erythematous lesions may not necessarily be red [20,21]. These differences could have resulted in lower (ie, less severe) clinician-rated VS signs scores in Black patients.

The differences we noted in PTS manifestations among Black participants should also be considered in the context of major disparities in access to health care and the quality of care delivered to these individuals in the United States. Multiple studies across decades have highlighted an unequal access to care for Black individuals, particularly with regard to ambulatory care and insurance coverage [22,23]. These barriers to care may have a tangible impact on the subsequent development of PTS after a DVT; for example, patients may not be able to access compression garments or anticoagulation medications, which could further predispose them to the development of PTS symptoms.

Another plausible explanation for the differences in VS symptoms and signs in Black and White participants in the ATTRACT trial could be the intervention itself (ie, PCDT). It is possible that certain racial groups benefited more from PCDT, which could explain the differences in VS scores we observed. However, analyses in the original ATTRACT trial did not demonstrate subgroup effects for benefit when self-reported race was categorized into White, Black, or Other [9]. Further, our analyses were adjusted for treatment group.

The strengths of this study lie in its well-characterized population of participants in a contemporary multicenter randomized controlled trial. Furthermore, to our knowledge, ours is the first study to evaluate the influence of self-reported race in the assessment of PTS, which remains above all a clinical diagnosis. Of note, studies that used other clinical scores such as the Venous Clinical Severity Score to assess chronic venous disease did not specifically report race as a demographic variable [24].

One of the major limitations of our study, as is seen in many clinical trials, is the greater numbers of White than Black participants, which may have reduced statistical power to detect correlations between signs and symptoms in the Black participants group. Furthermore, there were a certain proportion of participants (27, or 3%) who described self-reported race as ‘Other.’ Future studies should repeat similar analyses across different self-reported races. Finally, as the ATTRACT trial was involved multiple recruiting centers in various regions of the United States, there may have been heterogeneity in scoring on the part of the clinicians, and scoring of clinical VS signs in Black participants may have varied according to the participating clinicians’ familiarity with assessing PTS in patients of differing races.

## 4 Conclusion

Our study suggests that differences exist in PTS scores in self-identified Black vs. White patients and that individual signs and symptoms within the VS may have different utilities in different racial groups. It remains unclear whether these differences may have an impact on the overall utility of the VS in patients of varying racial backgrounds. Our findings highlight the need for more objective assessments of venous outcome measures that include cultural considerations.
