# Supplementary material for: Impact of self-reported race on Villalta Scale postthrombotic syndrome scores and correlation with venous disease-specific quality of life: an exploratory analysis of the Acute Venous Thrombosis: Thrombus Removal with Adjunctive Catheter-Directed Thrombolysis Trial
Source: Res Pract Thromb Haemost. 2024 Oct 29;8(8):102609. doi: 10.1016/j.rpth.2024.102609 (PMC11625206; doi:10.1016/j.rpth.2024.102609)
Supplement: Supplementary file 1 [file mmc1.zip › rpth_102609_Supplementary Table for Manuscript_mmc1.odt]

# Supplementary Table 1 : Villalta Scale Score with Missing Data by Self-reported Race*

| Self-reported Race | Raw | Imputed | Number of Subjects |
| --- | --- | --- | --- |
|  | Villalta Score, Symptoms: |  |  |
| Black | Cramps: |  |  |
| Missing | Missing | 22 |
| Missing | No/Minimal | 7 |
| Missing | Mild | 2 |
| Missing | Severe | 1 |
| White | Missing | Missing | 67 |
| Missing | No/Minimal | 7 |
| Missing | Mild | 4 |
| Missing | Severe | 1 |
| Black | Itching: |  |  |
| Missing | Missing | 22 |
| Missing | No/Minimal | 10 |
| White | Missing | Missing | 66 |
| Missing | No/Minimal | 10 |
| Missing | Mild | 1 |
| Missing | Moderate | 1 |
| Black | Pins and Needles: n (%) |  |  |
|  | Missing | Missing | 22 |
|  | Missing | No/Minimal | 8 |
|  | Missing | Mild | 2 |
| White | Missing | Missing | 66 |
|  | Missing | No/Minimal | 6 |
|  | Missing | Mild | 3 |
|  | Missing | Moderate | 3 |
| Black | Leg Heaviness: n (%) |  |  |
|  | Missing | Missing | 22 |
|  | Missing | No/Minimal | 8 |
|  | Missing | Mild | 1 |
| White | Missing | Missing | 66 |
|  | Missing | No/Minimal | 7 |
|  | Missing | Mild | 4 |
|  | Missing | Severe | 1 |
| Black | Pain: n (%) |  |  |
|  | Missing | Missing | 22 |
|  | Missing | No/Minimal | 7 |
|  | Missing | Mild | 2 |
| White | Missing | Missing | 66 |
|  | Missing | No/Minimal | 6 |
|  | Missing | Mild | 3 |
|  | Missing | Moderate | 2 |
|  | Missing | Severe | 1 |
|  | Villalta Score, Signs: |  |  |
| Black | Pretibial edema: |  |  |
| Missing | Missing | 23 |
| Missing | No/Minimal | 9 |
| Missing | Mild | 1 |
| Missing | Moderate | 1 |
| White | Missing | Missing | 68 |
| Missing | No/Minimal | 10 |
| Missing | Mild | 3 |
| Missing | Moderate | 1 |
| Black | Skin Induration: |  |  |
| Missing | Missing | 23 |
| Missing | No/Minimal | 11 |
| White | Missing | Missing | 698 |
| Missing | No/Minimal | 12 |
| Missing | Mild | 2 |
| Black | Hyperpigmentation |  |  |
| Missing | Missing | 24 |
| Missing | No/Minimal | 6 |
| Missing | Mild | 4 |
| Missing | Moderate | 1 |
| White | Missing | Missing | 68 |
| Missing | No/Minimal | 11 |
| Missing | Mild | 3 |
| Black | Venous ectasia: n (%) |  |  |
| Missing | Missing | 23 |
| Missing | No/Minimal | 10 |
| Missing | Mild | 1 |
| White | Missing | Missing | 68 |
| Missing | No/Minimal | 10 |
| Missing | Mild | 3 |
| Missing | Moderate | 1 |
| Black | Redness: |  |  |
| Missing | Missing | 23 |
| Missing | No/Minimal | 10 |
| Missing | Mild | 1 |
| White | Missing | Missing | 68 |
| Missing | No/Minimal | 12 |
| Missing | Mild | 2 |
| Black | Pain during Calf Compression: |  |  |
| Missing | Missing | 23 |
| Missing | No/Minimal | 13 |
| White | Missing | Missing | 68 |
| Missing | No/Minimal | 12 |
| Missing | Mild | 2 |

  * Patients with missing Villalta scores at 6 months were imputed by corresponding scores at 12 months
